# Antiparasitic Effect of Stilbene and Terphenyl Compounds against *Trypanosoma cruzi* Parasites

**DOI:** 10.3390/ph14111199

**Published:** 2021-11-22

**Authors:** Federica Bruno, Germano Castelli, Fabrizio Vitale, Simone Catanzaro, Valeria Vitale Badaco, Marinella Roberti, Claudia Colomba, Antonio Cascio, Manlio Tolomeo

**Affiliations:** 1 National Reference Center for Leishmaniasis (C.Re.Na.L.), Istituto Zooprofilattico Sperimentale della Sicilia, Via Gino Marinuzzi 3, 90129 Palermo, Italy; federica.bruno@izssicilia.it (F.B.); fabrizio.vitale@izssicilia.it (F.V.); simone.catanzaro87@gmail.com (S.C.); valeria.badaco@izssicilia.it (V.V.B.); 2 Department of Pharmacy and Biotechnology, University of Bologna, Via Belmeloro 6, 40126 Bologna, Italy; marinella.roberti@unibo.it; 3 Department of Health Promotion Sciences, Section of Infectious Diseases, University of Palermo, Via del Vespro 129, 90127 Palermo, Italy; claudia.colomba@libero.it (C.C.); antonio.cascio03@unipa.it (A.C.); mtolomeo@hotmail.com (M.T.)

**Keywords:** *Trypanosoma cruzi*, stilbene ST18, terphenyl TR4

## Abstract

Background: Chagas disease, also known as American trypanosomiasis, is a potentially life-threatening illness caused by the protozoan parasite *Trypanosoma cruzi*. No progress in the treatment of this pathology has been made since Nifurtimox was introduced more than fifty years ago, and this drug is considered very aggressive and may cause several adverse effects. This drug currently has severe limitations, including a high frequency of undesirable side effects and limited efficacy and availability, so research to discover new drugs for the treatment of Chagas disease is imperative. Many drugs available on the market are natural products as found in nature or compounds designed based on the structure and activity of these natural products. Methods: This study evaluated the in vitro antiparasitic activity of a series of previously synthesized stilbene and terphenyl compounds in *T. cruzi* epimastigotes and intracellular amastigotes. The action of the most selective compounds was investigated by flow cytometric analysis to evaluate the mechanism of cell death. The ability to induce apoptosis or caspase-1 inflammasomes was assayed in macrophages infected with *T. cruzi* after treatment, comparing it with that of Nifurtimox. Results: The stilbene ST18 was the most potent compound of the series. It was slightly less active than Nifurtimox in epimastigotes but most active in intracellular amastigotes. Compared to Nifurtimox, it was markedly less cytotoxic when tested in vitro on normal cells. ST18 was able to induce a marked increase in parasites positive for Annexin V and monodansylcadaverine. Moreover, ST18 induced the activation, in infected macrophages, of caspase-1, a conserved enzyme that plays a major role in controlling parasitemia, host survival and the onset of the adaptive immune response in Trypanosoma infection. Conclusions: The antiparasitic activity of ST18 together with its ability to activate caspase-1 in infected macrophages and its low toxicity toward normal cells makes this compound interesting for further clinical investigation.

## 1. Introduction

*Trypanosoma cruzi* (*T. cruzi*) is a protozoan parasite primarily transmitted by triatomine insects. It is the agent of Chagas disease, an endemic pathology in Latin America that affects about 6–8 million people worldwide [1] and causes approximately 50,000 deaths per year. The *T. cruzi* life cycles begin with insects sucking the blood of infected vertebrates with trypomastigote forms circulating in the bloodstream. Surviving trypomastigotes transform, after a few days, into a spherical stage, known as epimastigote stages. Epimastigotes migrate into the intestine where they divide intensely and are secreted by intestinal cells [2]. This intracellular pathogen invades a number of different cells, including macrophages and replicates within their cytoplasm. Macrophages, when inactivated, are susceptible to infection with trypomastigote forms of *T. cruzi*. Control of the parasite immediately after infection requires a robust inflammatory immune response. The acute phase ends when *T. cruzi* replication is suppressed by an effective T helper 1 cell response. However, infection persists in the absence of treatment, and failure to adequately down-regulate the inflammatory response appears to play a central role in the pathogenesis of chronic Chagas cardiomyopathy. The innate immune response against *T. cruzi* involves recruitment of the NLRP3 (nod-like receptor family pyrin domain containing 3) inflammasome with a caspase recruitment domain. These effects resulted in increased survival of the parasite within these macrophages, supporting the protective role of the inflammasome in infection control and protective role for the inflammasome in restricting parasite replication [3].

Only two nitroheterocyclic drugs, Nifurtimox and benznidazole, are available for the treatment of Chagas disease. These drugs have severe limitations, including a high frequency of undesirable side effects, long protocols for treatment and limited efficacy and availability, although they are effective for the treatment of acute infections. Experimental toxicity studies with Nifurtimox have evidenced neurotoxicity, testicular damage, ovarian toxicity and deleterious effects in adrenal, colonic, esophageal and mammary tissue, which frequently necessitate the cessation of treatment. In the case of benznidazole, deleterious effects on the adrenals, colon and esophagus have been observed. Both drugs exhibited significant mutagenic effects and were shown to be tumorigenic or carcinogenic in some studies [4,5]. Natural products have always been a source of a great variety of bioactive molecules, mostly substances from organisms’ secondary metabolism. Many drugs available on the market are natural products as found in nature or compounds designed based on the structure and activity of these natural products (semi-synthetic or completely synthetic) [6]. Recently, several natural and synthetic stilbenes and terphenyls have been studied for their anticancer and leishmanicidal properties [7,8,9,10]; in particular, we evaluated the antileishmanial activity of two compounds, a trans-stilbene derivative and a terphenyl derivative, namely, trans-1,3-dimethoxy-5-(4-methoxystyryl) benzene (ST18) and 3,4′′,5-trimethoxy-1,1′:2′,1′′-terphenyl (TR4), which presented the best activity and safety profiles [11,12].

In the current study, we evaluated the in vitro antiparasitic activity, in *T. cruzi* epimastigotes, of a series of cis- and trans-stilbene derivatives in which a variety of substituents were introduced at positions 2′, 3′ and 4′ of the stilbene scaffold, while the 3,5-dimethoxy motif was maintained. Additionally, we studied a series of terphenyl compounds incorporating a phenyl ring as a bioisosteric substitution of the stilbene alkenyl bridge that could enable the discovery of a natural product-based drug.

We observed that the stilbene ST18 was endowed with potent antiparasitic activity in both *T. cruzi* epimastigotes and intracellular *T. cruzi* amastigotes. Compared to Nifurtimox, it was markedly less cytotoxic when tested in vitro on normal and differentiated cells. Moreover, this compound induced the activation, in infected macrophages, of caspase-1, an evolutionarily conserved enzyme that plays a major role in controlling parasitemia, host survival and the onset of the adaptive immune response in *T. cruzi* infection.

## 2. Results

### 2.1. Anti-Trypanosoma cruzi Activity

Table 1 shows the in vitro antiparasitic effects evaluated as the IC_50_s of different stilbenes (**ST18**, 1–10) and terphenyls (**TR4**, 11–15) in *T. cruzi* epimastigotes.

**Figure 1:**
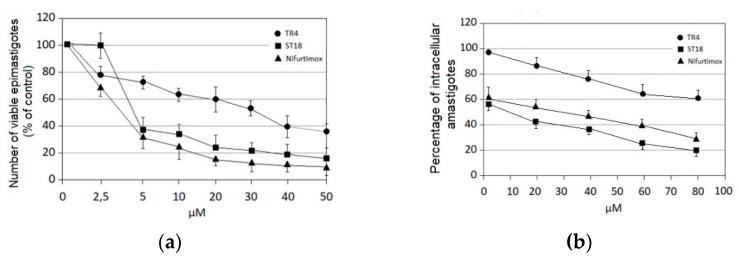
Effects of the compounds Nifurtimox, ST18 and TR4 in *Trypanosoma cruzi* epimastigotes and intracellular amastigotes. (**a**) Number of viable *T. cruzi* epimastigotes expressed as percentage of untreated control after 72 h exposure to increasing concentrations of Nifurtimox, ST18 and TR4. (**b**) Number of intracellular amastigotes expressed as percentage of the untreated control after 72 h treatment with Nifurtimox, ST18 and TR4. Bars indicate the means ± SEs from four independent experiments. Data obtained are statistically significant at *p* < 0.05.

These compounds were previously synthesized by us, except ST18 and 6, which were reported by Kim et al. [13]. The data were compared to those obtained with Nifurtimox, the drug currently used for the treatment of *T. cruzi* infection. The most active compounds of the series were the stilbene ST18 (IC_50_ = 4.6 µM) and the terphenyl TR4 (IC_50_ = 30 µM). Figure 1 shows the in vitro effects of Nifurtimox, ST18 and TR4 used at increasing concentrations for 72 h in *T. cruzi* epimastigotes. ST18 was markedly more potent than TR4 but less active than Nifurtimox. Upon entering the mammalian host, *T. cruzi* parasites transform into the amastigote stage, residing inside the phagolysosomal vacuoles of macrophages. We evaluated the anti-amastigote efficacy in differentiated macrophage cells (derived from U937 cells) infected with *T. cruzi*, as reported in the Materials and Methods. Infected macrophages were treated with Nifurtimox, ST18 and TR4 at increasing concentrations for 72 h. Differently from the results obtained in epimastigotes, the antiparasitic effect of ST18 in infected macrophages was higher than that observed using Nifurtimox.

### 2.2. Mammalian Cell Cytotoxicity and SI

**Figure 2:**
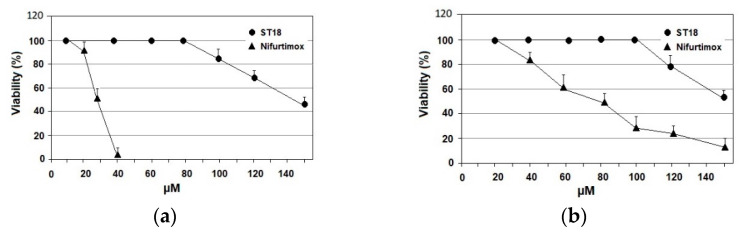
Cytotoxic effects of compounds ST18 and Nifurtimox in mammalian cells. (**a**) Cytotoxic effects of compounds ST18 and Nifurtimox in primary epithelial cells of Cercopiteco (CPE). (**b**) Cytotoxic effects of compounds ST18 and Nifurtimox in U937 macrophage cells. Bars indicate the means ± SEs from four independent experiments. Data obtained are statistically significant at *p* < 0.05.

Primary epithelial cells of Cercopiteco (CPE) and macrophages derived by the differentiation of U937 cells were treated with increasing concentrations of ST18 and Nifurtimox. The cytotoxicity was evaluated after 72 h through the MTT assay. ST18 showed very low cytotoxicity in both cell lines compared to Nifurtimox. In macrophages, the IC_50_ of ST18 was 143 µM, while the IC_50_ of Nifurtimox was 28 µM, with an SI of 31 for ST18 and 8.75 for Nifurtimox. In CPE, the IC_50_s of ST18 and Nifurtimox were 155 and 77 µM, respectively, with an SI of 33.7 for ST18 and 24 for Nifurtimox. (Figure 2).

### 2.3. Cell Cycle

**Figure 3:**
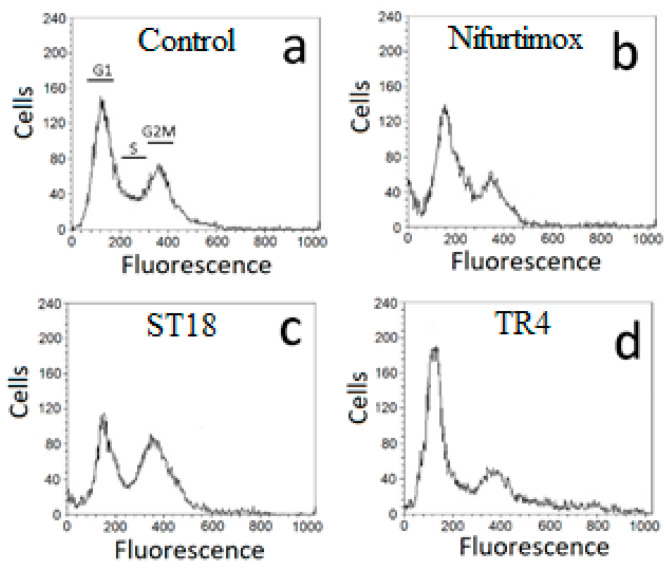
Effects of Nifurtimox, ST18 and TR4 on DNA content in/number of *Trypanosoma cruzi* epimastigotes. The parasites were cultured without compound (control, Panel (**a**) or with 35 µM Nifurtimox (Panel (**b**), 50 µM ST18 (Panel (**c**) and 90 µM TR4 (Panel (**d**). Cell cycle distribution was analyzed using the standard propidium iodide procedure. G1, S and G2–M cells are indicated in (Panel (**a**)).

The effects of Nifurtimox, ST18 and TR4 on the cell cycle distribution of *T. cruzi* were analyzed using a FACScan flow cytometer. To exclude dead cells that are often located in a sub-G0–G1 peak in the study of the cell cycle, we decided to study the effects of each compound on the cell cycle by treating the parasites for a period of time and with concentrations of each compound that caused a block of cell growth (evaluated by counting the parasites in a hemocytometer) without causing a relevant number of dead cells (evaluated by trypan blue staining). Since, after 72 h of treatment, the cell growth inhibition was associated with an increase in cell death (data not shown), we studied the effects of each compound on the cell cycle after only 48 h of drug exposure, treating the parasites with 35 µM Nifurtimox, 50 µM ST18 and 90 µM TR4. This treatment caused a complete block of cell growth, with a percentage of dead cells lower than 10%. The cell cycle distribution was analyzed using the standard propidium iodide procedure. Nifurtimox did not result in important variations in the cell cycle distribution, but caused a slight reduction in the G2M peak. By contrast, ST18 caused an evident block in G2M, while TR4 resulted in a block in G1 (Figure 3).

### 2.4. Flow Cytometry Analysis of Physical Parameters (Cell Size and Granularity)

**Figure 4:**
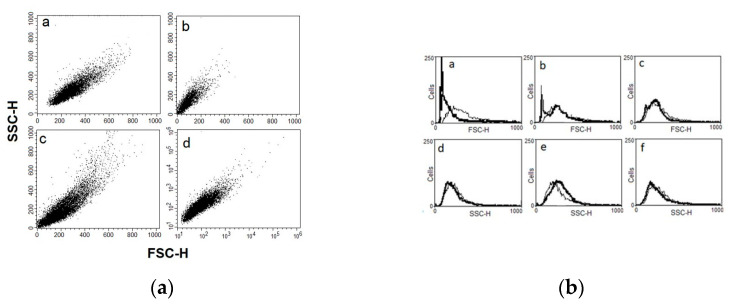
FACS analysis of *Trypanosoma cruzi* epimastigotes cell volume populations. (**a**) Forward light scatter (FSC-H) was considered as a function of cell size, and side light scatter (SSC-H), as a result of cell granularity. Density plots for FSC versus SSC in *T. cruzi* epimastigotes after 72 h treatment with Nifurtimox (Panel (b)), ST18 (Panel (c)) and TR4 (Panel (d)). Untreated control is represented in Panel (a). (**b**) Representative FACS histogram showing FSC-H and SSC-H of *T. cruzi* epimastigotes after 72 h treatment with Nifurtimox (Panels (a) and (d)) ST18 (Panels (b) and (e)) and TR4 (Panels (c) and (f)). Thin line: Non-treated control parasites; thick line: Parasites treated with each compound. Data are representative of three separate experiments.

We studied the physical parameters of *T. cruzi* parasites treated with Nifurtimox, ST18 and TR4 using a FACScan flow cytometer as previously reported by Jimenez et al. [14]. Figure 4a shows density plots for forward scatter (FSC) versus side scatter (SSC) in *T. cruzi* epimastigotes untreated or treated with 35 µM Nifurtimox, 50 µM ST18 and 90 µM TR4 for 72 h. The measurement of forward scatter allows for the discrimination of cells by size. The FSC intensity is proportional to the diameter of the cell. Side scatter measurement provides information about the internal complexity (i.e., granularity) of a cell. The analysis of the density plot of Trypanosome epimastigotes treated with Nifurtimox shows a marked reduction in the average cell size compared to the control. By contrast, the FACS analysis of Trypanosome epimastigotes treated with ST18 shows a heterogeneous population characterized by parasites with low dimension and parasites with increased size and granularity. No important modifications were observed with TR4. These data are confirmed by the FACS histograms as shown in Figure 4b.

### 2.5. Annexin V and MDC Labeling

**Figure 5:**
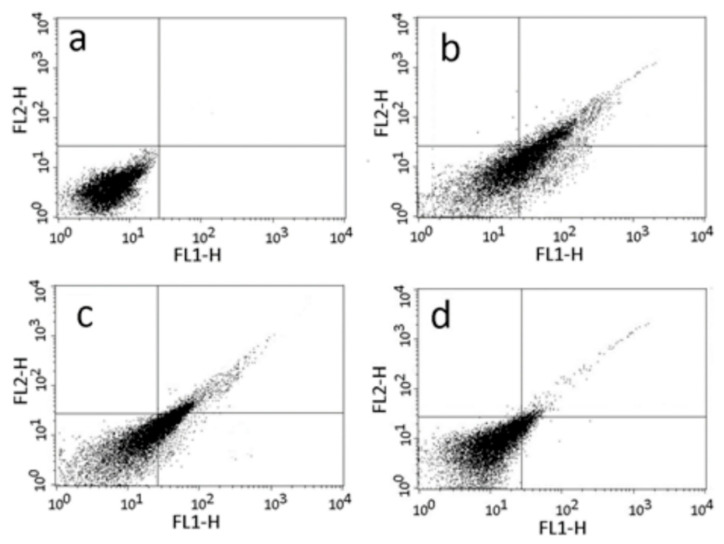
Analysis of phosphatidylserine (PS) extracellular exposure. Representative dot plot of FACS analysis for PS exposure, measured by double staining with Annexin V-FITC and propidium iodide (PI) in *T. cruzi* epimastigotes after 72 h treatment with Nifurtimox (Panel **b**), ST18 (Panel **c**) and TR4 (Panel **d**). Non-treated control is represented in (Panel **a**). Lower-left quadrant represents control cells (Annexin V negative/PI negative), lower-right quadrant represents early apoptotic cells (Annexin V positive/PI negative), upper-right quadrant represents late apoptotic cells (Annexin V positive/PI positive) and upper-left quadrant represents necrotic cells (Annexin V negative/PI positive). Data are representative of three separate experiments.

The loss of cell volume or cell shrinkage is a hallmark of the early phase of the apoptotic process. In order to confirm whether the volume reduction of parasites was related to apoptosis, the exposure of phosphatidylserine at the cell surface was analyzed by an Annexin V labeling test after treatment with Nifurtimox, ST18 and TR4. A significant increase in the percentage of parasites positive for Annexin V was observed after treatment with Nifurtimox and, to a lesser extent, after treatment with ST18 (Figure 5).

**Figure 6:**
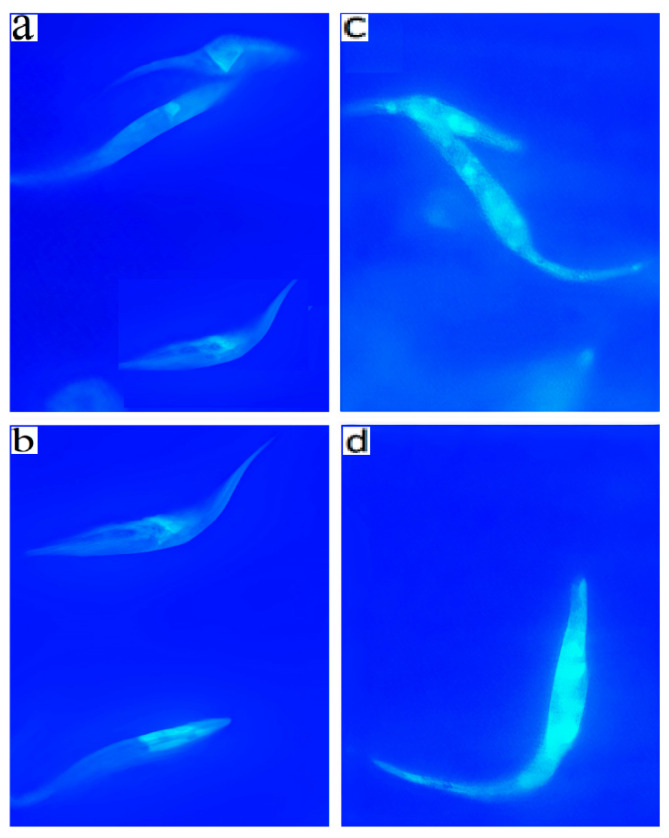
Autophagic induction by ST18 in *Trypanosoma cruzi* epimastigotes. Parasites were incubated with 0.05 mM MDC in PBS at 37 °C for 10 min and observed under a fluorescent microscope, Nikon Eclipse E 200 (100×). (**a**,**b**): Control. (**c**,**d**): *T. cruzi* epimastigotes treated for 72 h with 40 µM ST18. Eclipse E 200 (100×). a and b: Control. (**c**,**d**): *T. cruzi* epimastigotes treated for 72 h with 40 µM ST18.

Since the analysis of the physical parameters of *T. cruzi* treated with ST18 also showed a cell population with increased size and granularity, parameters that are hallmarks of the autophagic process, the parasites were treated with monodansylcadaverine (MDC), a specific fluorescent marker for autophagic vacuoles [15]. About 30% of the parasites treated for 72 h with 40 µM ST18 were strongly positive in the MDC test, showing numerous fluorescent vacuoles in the cytoplasm. These vacuoles were not observed in the untreated control and in samples treated with Nifurtimox or TR4 (data not shown) (Figure 6).

### 2.6. Caspase-1

**Figure 7:**
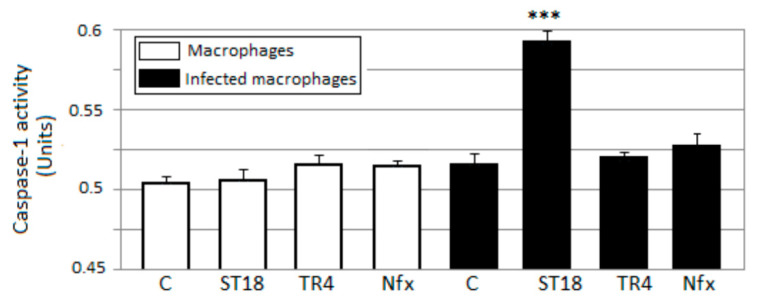
Caspase-1 activity. Levels of active caspase-1 in U937 macrophages infected with *T. cruzi* epimastigotes after 48 h of treatment with 50 µM ST18, TR4 and Nifurtimox (Nfx). C = untreated control. Bars indicate the means ± SEs from four independent experiments. *** *p* < 0.05 vs. control.

Infection with *T. cruzi* results in the activation of caspase-1 and inflammasome formation. The inflammasome is indispensable for controlling parasitemia, host survival and the onset of the adaptive immune response [3]. In this context, inflammasome activation is fully dependent on caspase-1. We evaluated the levels of active caspase-1 in U937 macrophages infected with *T. cruzi* after treatment with Nifurtimox, ST18 and TR4. In macrophages infected with trypanosomes and treated with ST18, spectrophotometric analysis showed a substantial increase in active caspase-1 compared to the control. By contrast, no increase in caspase-1 was observed in samples of infected macrophages treated with Nifurtimox or TR4 or in uninfected macrophages treated with ST18 (Figure 7).

## 3. Discussion

We evaluated the in vitro antiparasitic effects, in *T. cruzi* epimastigotes, of a series of cis- and trans-stilbenes bearing a 3,5-dimethoxy motif at the A phenyl ring and an amino, methoxy and hydroxyl function at the 2′, 3′- and/or 4′-positions at the B phenyl ring. Moreover, in an attempt to increase the chemical diversity of the compounds, we studied a small series of terphenyl derivatives that notably do not bear the ethylene double bond that is the main reason for the chemical and metabolic instability of stilbenes [3,15]. The data were compared to those obtained with Nifurtimox, which is the drug currently used for the treatment of Trypanosome infections. Among the stilbene series, ST18 bearing a 4′-methoxy function was the most active compound, showing an IC_50_ of 4.6 ± 0.4 µM. Regarding the terphenyl derivatives, the best results were obtained with the trimethoxylated compound TR4 (IC_50_ = 30 ± 4.3), which is an ortho-terphenyl analogue of ST18. Nifurtimox was more active than ST18 in *T. cruzi* epimastigotes but less active in intramacrophagic *T. cruzi* amastigotes.

The most interesting data observed in this study were the difference in the selectivity index values between ST18 and Nifurtimox. Nifurtimox is a drug with several adverse effects including mutagenic and tumorigenic effects [5]. ST18 has been described in the literature by different names, including resveratrol trimethyl ether (RTE) [16,17], MR-3 [18,19], M-5 [20], BTM-0521 [21], trimethoxy resveratrol [22], trimethylated resveratrol [23] and TMS [17,24]. It is a natural stilbene isolated from *Virola cuspidata* and *Virola elongata* bark [24,25]. Natural stilbenes have received increasing attention due to their potent antioxidant properties and their marked effects in the prevention of various oxidative-stress-associated diseases such as cancer [25]. A number of clinical trials using natural stilbenes such as resveratrol and pterostilbene have shown that they are therapeutically effective and pharmacologically safe because they show no organ-specific or systemic toxicity [26,27,28,29,30]. Preclinical pharmacokinetic studies have shown that ST18 has appropriate pharmacokinetic profiles that make it a promising drug candidate for further pharmaceutical development [16]. It exhibited anti-proliferative and/or apoptosis-inductive activities in various cancer cells, with a potency usually higher than that of resveratrol [17,20,31,32,33]. Moreover, it has shown anti-inflammatory [34,35,36,37], gastroprotective [38] and hepatoprotective activities [23]. Here, we have demonstrated that ST18 showed very low toxicity toward monocytic and macrophagic cells, and the SI for *T. cruzi* parasites was higher than that calculated for Nifurtimox.

Several studies have shown that Nifurtimox induces the production of reactive oxygen species (ROS) and subsequent apoptosis in neoplastic cells [39,40,41]. While programmed cell death is very controversial in unicellular eukaryotes, we observed that Nifurtimox caused a marked reduction in the average cell size of *T. cruzi* epimastigotes and a significant increase in the percentage of parasites positive for Annexin V. This compound did not cause, in the parasites, an increase in MDC, an important marker of autophagy. By contrast, ST18 produced a heterogeneous population characterized by parasites with low dimension and parasites with increased size and granularity. ST18 induced an increase in both Annexin V- and MDC-positive parasites.

Several studies have reported the activation of the autophagic process in Trypanosomatids during starvation responses and lifecycle developments. Moreover, endoplasmic reticulum (ER) stress and antiparasitic drugs can induce autophagy in *T. brucei* and *T. cruzi* [42,43,44]. In our experiments, ST18 caused both phosphatidylserine expression and dansylcadaverina staining in *T. cruzi*, suggesting that this compound could be capable of activating both apoptosis and autophagy.

Lim et al. [45] obtained similar results in *T. brucei* rhodesiense using two piperidine alkaloids, (+)-spectaline and iso-6-spectaline. These compounds caused the formation of autophagic vacuoles that were susceptible to monodansylcadaverine staining, indicating the activation of the autophagic process. When trypanosomes were treated with piperidine alkaloids for 72 h, they showed apoptotic aspects, including phosphatidylserine exposure.

Several studies have demonstrated that autophagy and apoptosis communicate with each other to decide the fate of the cell during physiological and pathological conditions [46]. It has been supposed that, after the activation of stress or drug-induced autophagy, when the stress condition increases towards a point of no return, cells block autophagy and activate programmed cell death. Interestingly, the analysis of the cell cycle showed that both Nifurtimox and TR4 caused a decrease in parasites in the G2M phase of the cell cycle, while ST18 resulted in an important block in G2M. A correlation between G2M block and autophagy activation has been observed in different experimental models, but the precise mechanism by which microtubule-targeting agents induce autophagic cell death is not known [47,48,49,50].

Finally, we observed that ST18, but not TR4 and Nifurtimox, induced a marked increase in active caspase-1 in *T. cruzi*-infected macrophages. The capability of ST18 to activate caspase-1 in *T. cruzi*-infected macrophages may, in part, explain the greater antiparasitic effect of ST18 than Nifurtimox in intramacrophagic trypanosomes. In fact, Yu et al. [51] demonstrated that canonical inflammasome activation triggers ROS production in macrophages in a caspase-1-dependent manner. Reactive oxygen species (ROS) protect the host against a large number of pathogenic microorganisms including trypanosomes [52,53].

## 4. Materials and Methods

### 4.1. Parasite Cultures

A strain of *T. cruzi* taken from a stock archive of the OIE Reference Laboratory National Reference Center for Leishmaniasis (C.Re.Na.L. Palermo, Italy) was cultured in 25 cm^2^ flasks (Falcon) at 25 °C and pH 7.18 in RPMI-PY medium, which consisted of RPMI 1640 (Sigma R0883) supplemented with an equal volume of Pepton-yeast medium, 10% fetal bovine serum (FBS), 1% glutamine, 250 μg/mL gentamicin and 500 μg/mL 5-fluorocytosine [54].

### 4.2. Compound and Sample Preparation

The compounds ST18 and 6 were synthesized as reported by Kim et al. [13]; compounds **1**–**5** and **8**–**10** were prepared as previously described by us [8]; compounds 7, TR4 and 13–14 were prepared as previously described by us [9]; 15 was synthesized as reported by Pizzirani et al. [7]. The purity of the compounds was determined by elemental analyses and was ≥97%. Each compound was dissolved in dimethyl sulfoxide (DMSO) to make a stock solution at a concentration of 20 mM, stored at −20 °C and protected from light. In each experiment, the DMSO never exceeded 0.2%, a percentage that did not interfere with cell growth. Nifurtimox was obtained from Merck Sigma-Aldrich (Milano, Italy).

### 4.3. Epimastigote Viability Assay

To evaluate the effects of compounds in cultures of *T. cruzi*, a viability assay protocol similar to that described by Castelli et al. [11] was used with some modifications. Exponentially growing *T. cruzi* were dispensed at a concentration of 4 × 10^6^/mL in 25 m^2^ flasks (Falcon) and treated with increasing concentrations (from 1 to 200 μM) of each compound. After 72 h of treatment, the parasites were centrifugated and resuspended in 1 mL of RPMI-PY medium. The suspension of *T. cruzi* from each treatment was mixed with 0.4% trypan blue solution at a ratio of 3:1 (*v/v*). The percentage vitality of *T. cruzi* was observed by counting in a Bürker hemocytometer for the enumeration of stained and unstained cells, taken respectively as the dead and living cells, in comparison with those for the control culture (100% viability). The IC_50_ (half maximal inhibitory concentration) was evaluated after 72 h and was calculated by regression analysis (GraphPad software).

### 4.4. Effects of Compounds in Intracellular Amastigotes

U937 monocytic cells (1 × 10^5^ cells/mL) in the logarithmic phase of growth were plated onto chamber Lab Tek culture slides in 2.5 mL of RPMI 1640 (Sigma) 10% FBS medium containing 25 ng/mL of phorbol 12-myristate 13-acetate (Sigma) for 18 h to induce macrophage differentiation.

After incubation, the medium was removed by washing twice with RPMI 1640 medium. Non-adherent cells were removed and the macrophages were further incubated overnight in RPMI 1640 medium supplemented with 10% FBS. Then, the adherent macrophages were infected with *T. cruzi* epimastigotes at a parasite/macrophage ratio of 50:1 for 24 h at 37 °C in 5% CO_2_. Free epimastigotes were removed by three extensive washes with RPMI 1640 medium, and the infected macrophages were either incubated for 48 h in medium alone (control) or incubated with Nifurtimox, ST18 or TR4. To stain intracellular amastigotes, cells were fixed with iced methanol to permeabilize the cell membranes to ethidium bromide and stained with 100 µg/mL ethidium bromide. The number of amastigotes was determined by examining three coverslips for each treatment. At least 200 macrophages were counted by visual examination under 400× magnification using a fluorescence microscope, Nikon Eclipse E200 (Nikon Instruments Europe, Amsterdam, The Netherlands), equipped with a green filter, to determine the number of intracellular amastigotes. The number of intracellular amastigotes in the samples treated with each compound was expressed as a percentage of the untreated control.

### 4.5. Mammalian Cell Cytotoxicity

The potential cytotoxic action of each compound was checked using the 3-(4,5-dimethylthiazol-2-yl)-2,5-diphenylterazolium bromide (MTT) assay on macrophages derived from U937 cells and in primary epithelial cells of Cercopiteco (CPE). Macrophages and CPE cells were cultured in RPMI 1640 (Sigma) supplemented with 10% fetal bovine serum (FBS, Gibco), penicillin (100 IU/mL) and streptomycin (100 mg/mL). The cells were grown at 37 °C in 5% CO_2_ and passaged twice a week. In each experiment, the cells (10^5^/well) were incubated in 96-well plates overnight in a humidified 5% CO_2_ atmosphere at 37 °C to ensure cell adherence. After 24 h, the cells were treated with increasing concentrations of each compound. Non-treated cells were included as a negative control. After 72 h of incubation with each compound, the MTT (5 mg/mL) was added to each well and incubated at 37 °C for 4 h. Then, the medium and MTT were removed, the cells were washed using PBS and 200 µL of DMSO was added to dissolve the formazan crystals. The absorbance was measured using a microplate reader Spectrostar Nano (BMG LabTech) at 570 nm. The reduction of MTT to insoluble formazan was performed by the mitochondrial enzymes of the viable cells and so was an indicator of cell viability. Therefore, decreases in absorbance indicate toxicity to the cells. The viability was calculated using the following formula: [(L2/L1) × 100], where L1 is the absorbance of the control cells and L2 is the absorbance of the treated cells. The IC_50_ was calculated by regression analysis (GraphPad software).

The selectivity index (SI) was determined by dividing the IC_50_ calculated for the mammalian cells by the IC_50_ calculated for the *T. cruzi* parasites.

### 4.6. Cell Cycle Analysis by Flow Cytometry

Epimastigotes (4 × 10^6^) were incubated for 48 h with each compound at 26 °C. Afterward, the parasites were washed 3 times with PBS containing 0.02 M EDTA to avoid clumps and were then fixed with cold methanol for 24 h. The parasites were resuspended in 0.5 mL of PBS containing RNase I (50 µg/mL) and PI (25 µg/mL) and were then incubated at 25 °C for 20 min. The material was kept on ice until analysis. The stained parasites were analyzed using single-parameter frequency histograms by using a FACScan flow cytometer (Becton Dickinson, San Jose, CA, USA).

### 4.7. Cell Volume Determination

Epimastigotes were collected by centrifugation at 1000× *g*, washed twice in PBS, resuspended in PBS to 500 × 10^3^ parasites/mL and analyzed using a FACScan flow cytometer (Becton Dickinson, San Jose, CA, USA). Density plots of the forward (FSC) versus side (SSC) scatter represent the acquisition of 10 × 10^3^ events.

### 4.8. Determination of Apoptosis by Annexin V

The externalization of phosphatidylserine on the outer membranes of the parasites with and without treatment was determined by using an Annexin V labeling kit following the manufacturer’s protocol (Annexin-V-FITC Apoptosis Detection Kit Alexis, Switzerland). Briefly, epimastigotes (2 × 10^6^) were washed with PBS and centrifuged at 500× *g* for 5 min. The pellet was suspended in 100 µL of staining solution containing FITC-conjugated Annexin V and propidium iodide (Annexin-V-Fluos Staining Kit, Roche Molecular Biochemicals, Germany) and incubated for 15 min at 20 °C. The Annexin V-positive parasites were determined by using a FACScan flow cytometer (Becton Dickinson, San Jose, CA, USA).

### 4.9. Monodansylcadaverine Labelling

Monodansylcadaverine (MDC), which is an autofluorescent compound due to the dansyl residue conjugated to cadaverine, has been shown to accumulate in acidic autophagic vacuoles. The concentration of MDC in an autophagic vacuole is the consequence of an ion-trapping mechanism and an interaction with lipids in autophagic vacuoles (autophagic vacuoles are rich in membrane lipids). The use of MDC staining is a rapid and convenient approach by which to assay autophagy, as shown in cultured cells [15]. Autophagic vacuoles were labeled with MDC by incubating cells on coverslips with 0.05 mM MDC in PBS at 37 °C for 10 min. After incubation, the cells were washed four times with PBS and immediately analyzed by fluorescence microscopy (Nikon Eclipse E 200, Japan) equipped with a blue filter. Images were obtained with a Nikon Digital Sight DS-SM (Nikon, Japan) camera and processed using the program EclipseNet, version 1.20.0 (Nikon, Japan).

### 4.10. Caspase-1 Detection

To evaluate the level of active caspase-1, the U937 cell line in macrophagic form infected with *T. cruzi* was used. Infected macrophages were incubated for 24 h at 37 °C in 5% CO_2_. Free parasites were removed by extensive washing with RPMI 1640 medium, and infected cells were either incubated in medium alone (infection control) or incubated with each compound. After 48 h, the culture medium was removed and treated with the caspase-1 assay kit (Promega) following the manufacturer’s instructions.

### 4.11. Statistical Analysis

All the assays were performed by two observers with three replicate samples and repeated with three new batches of parasites. The means and standard errors of at least three experiments were determined. The differences between the mean values obtained for the experimental groups were evaluated using Student’s *t* test. *p*-values of 0.05 or less were considered significant. The entire statistical analysis was performed using the GraphPad Prism 5 software. The IC_50_ values were calculated by linear regression.

## 5. Conclusions

In conclusion, after testing 17 different compounds previously designed and synthesized by us, we selected a stilbene compound, ST18, endowed with potent antiparasitic activity in *T. cruzi* epimastigotes and intracellular amastigotes. The antiparasitic activity of ST18 together with its ability to activate caspase-1 in infected macrophages and its low toxicity toward normal cells makes this compound interesting for further biological and clinical studies in *T. cruzi*.

## Figures and Tables

**Table 1:** IC_50_ values of stilbenes (**ST18**, 1–10), terphenyls (**TR4**, 11–15) and Nifurtimox in *Trypanosoma cruzi* epimastigotes.

Compound	Structure	IC_50_^1^ (µM) ± SE ^2^
**ST18**	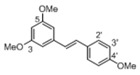	4.6 ± 0.4
**1**	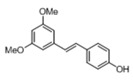	34 ± 5.2
**2**	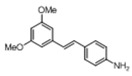	>50
**3**	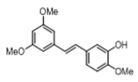	>50
**4**	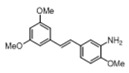	>50
**5**	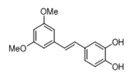	38 ± 5
**6**	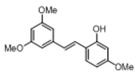	>50
**7**	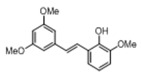	>50
**8**	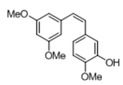	48 ± 6.8
**9**	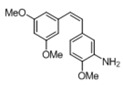	33 ± 4.9
**10**	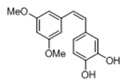	>50
**TR4**	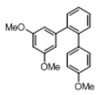	30 ± 4.3
**11**	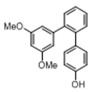	>50
**12**	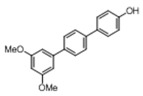	34 ± 5.8
**13**	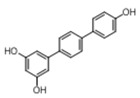	46 ± 6.3
**14**	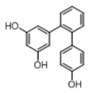	>50
**15**	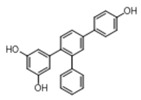	>50
**Nifurtimox**	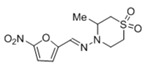	± 0.4

^1^ IC_50_, half maximal inhibitory concentration; ^2^ SE, standard error.

## Data Availability

The data are available at bioRxiv preprints; doi: https://doi.org/10.1101/2021.02.23.432446 (accessed on 23 February 2021).
